# FUNDC1-induced mitophagy protects spinal cord neurons against ischemic injury

**DOI:** 10.1038/s41420-023-01780-9

**Published:** 2024-01-05

**Authors:** Dehui Chen, Linquan Zhou, Gang Chen, Taotao Lin, Jiemin Lin, Xin Zhao, Wenwen Li, Shengyu Guo, Rongcan Wu, Zhenyu Wang, Wenge Liu

**Affiliations:** 1 Department of Orthopedics, Fujian Medical University Union Hospital, Fuzhou, 350001 Fujian China; 2 School of Health, Fujian Medical University, Fuzhou, 350001 Fujian China

**Keywords:** Cell death in the nervous system, Mitophagy, Spinal cord injury

## Abstract

Local ischemia and hypoxia are the most important pathological processes in the early phase of secondary spinal cord injury (SCI), in which mitochondria are the main target of ischemic injury. Mitochondrial autophagy, also known as mitophagy, acts as a selective autophagy that specifically identifies and degrades damaged mitochondria, thereby reducing mitochondria-dependent apoptosis. Accumulating evidence shows that the mitophagy receptor, FUN14 domain-containing 1 (FUNDC1), plays an important role in ischemic injury, but the role of FUNDC1 in SCI has not been reported. In this study, we aimed to investigate whether FUNDC1 can enhance mitophagy and inhibit neuronal apoptosis in the early stage of SCI. In a rat SCI model, we found that FUNDC1 overexpression enhanced neuronal autophagy and decreased neuronal apoptosis in the early stage of injury, thereby reducing spinal cord damage. In vitro studies showed that the neuroprotective effects of FUNDC1 were achieved by inhibiting mitochondria-dependent apoptosis and improving mitochondrial function. In addition, FUNDC1 enhanced mitophagy. The protective effects of FUNDC1 against apoptosis and mitochondrial dysfunction were reversed by 3-methyladenine (3-MA), an autophagy inhibitor. Taken together, our results confirm that FUNDC1 can protect against neuronal loss after SCI by inducing mitophagy, inhibiting mitochondria-dependent apoptosis, and improving mitochondrial function.

## Introduction

Spinal cord injury (SCI) is a devastating disease that can cause temporary or permanent irreversible and severe sensory and motor impairment, which currently has no cure. SCI often causes severe physical, psychological, and economic burdens to the patient’s family [1].

SCI can be divided into primary and secondary injuries. Primary injury is irreversible and caused by direct physical force, including spinal cord compression, contusion, and shear injury. In contrast, secondary injury is progressive and secondary to mechanical damage to the nerves and vascular tissues, causing local ischemia, hypoxia, free radical production, excitotoxicity, electrolyte imbalance, and inflammatory response, resulting in a series of complex pathological processes such as secondary cell death or necrosis. Local ischemia and hypoxia are the most important pathological processes in secondary injury [2–4]. The secondary injury is controllable; however, it can cause severe morbidities. Neuronal death is a key factor in secondary injury and a major cause of neurological dysfunction. Apoptosis is an important type of neuronal death in SCI [5]. Therefore, preventive strategies are essential for secondary injuries, especially to reduce secondary neuronal apoptosis.

Mitochondria are widely distributed in spinal cord neurons and play key roles in various cellular processes, including ATP production, maintenance of Ca2+ homeostasis, reactive oxygen species (ROS) production, and neuronal apoptosis [6]. However, the accumulation of damaged mitochondria interferes with normal cell function and may lead to uncontrolled oxidative stress, excessive inflammatory response, or amplified apoptosis through increased ROS production and mislocalized mitochondrial DNAs (mtDNAS) [7–9]. Therefore, to ensure normal mitochondrial function, their quality must be closely monitored. Mitochondrial autophagy, also known as mitophagy, is a selective autophagic process that specifically identifies and degrades the damaged mitochondria, allowing their components to be reused. Mitophagy also prevents the accumulation of mtDNA mutations and reprograms cellular metabolism, thereby reducing cell death [10, 11]. Mitophagy plays an important role in SCI, but the exact mechanism is not known. FUN14 domain-containing 1 (FUNDC1), a mitochondrial outer membrane protein, was first reported as a novel hypoxia-induced mitophagy receptor in 2012 [12]. It has a classical LC3 interaction region (LIR) and can recruit LC3 to mitochondria under ischemia and hypoxia, thus inducing mitophagy [13].

In recent years, many studies have found that FUNDC1 is closely associated with the progression of ischemic and metabolic diseases and cancer [14]. In particular, previous studies have shown that FUNDC1 plays a crucial role in the development or treatment of myocardial, renal, and intestinal ischemic injury [15–18]. However, little is known about the role of FUNDC1 in SCI. Therefore, we investigated whether FUNDC1 plays a neuroprotective role during the early stage of SCI in vitro and in vivo. We also explored the relationship between FUNDC1 and autophagy, apoptosis, and mitochondrial dysfunction in SCI, which may provide a potential therapeutic strategy for early intervention in SCI.

## Results

### Early activation of FUNDC1 protected against SCI and promoted recovery from SCI

**Fig. 1:**
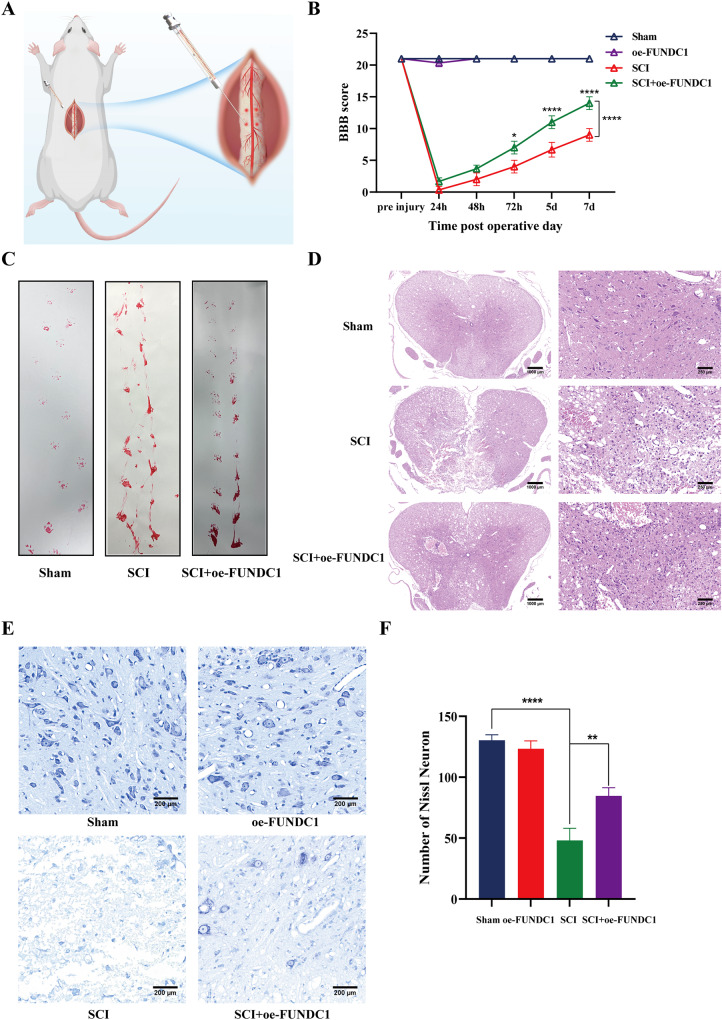
Early activation of FUNDC1 protected against SCI and promote the repair process. **A** The schematic diagram for local injection of AAV-Fundc1 virus into the spinal cord. **B** BBB scores at various times following spinal cord contusions in each group of rats (mean ± SD, *n* = 3). **C** Example footprints of rats walking 1 week following SCI (*n* = 3). Red: paw print—hind paw. **D** Representative cross-sectional H&E staining of the spinal cord for each group (*n* = 3). Scale bar = 1000 μm or 200 μm. **E**, **F** Nissl staining was performed to quantify the number of surviving neurons under each field of view in each group (mean ± SD, *n* = 3). Scale bar = 250 μm. Two-way ANOVA was used to compare the groups. ****p < 0.0001, **p < 0.01, and *p < 0.05.

AAV-DJ has become a highly efficient recombinant AAV vector recognized worldwide. It is often used for gene editing in the central nervous system due to its efficacy in transfecting the nervous system and its preferential transfection of neurons after direct injection into the brain and spinal cord [19–21]. Therefore, we induced local overexpression of FUNDC1 by injecting the AAV virus into the rat spinal cord (Fig. 1A). Proteins were extracted from spinal cord tissue after 7 days.

We carried out Basso-Beatie-Bresnahan (BBB) scoring at 7 days following surgery to explore the therapeutic effect of overexpression of FUNDC1 on locomotion recovery in the early stage after SCI. At 24 and 48 h after injury, no significant differences in BBB scores appeared between the SCI and SCI+oe-FUNDC1 groups. The BBB scores were higher in the SCI+oe-FUNDC1 group compared to the SCI group from 72 h to 7 days after injury (Fig. 1B). Furthermore, footprints were analyzed manually to evaluate the gait. Compared with the sham group, all animals showed a significant decrease in hindlimb motor function. The animals in the oe-FUNDC1 + SCI group showed significant recovery in their gait and motor coordination compared with the SCI group at 1 week after contusion (Fig. 1C).

Then, H&E staining of spinal cord tissues 1 week after SCI showed significant disorganization of tissue structure and extensive destruction in the SCI group compared with the sham group. However, tissue structure disorganization was less severe in the SCI+oe-FUNDC1 group compared with the SCI group (Fig. 1D).

We analyzed the survival of spinal cord anterior horn neurons by Nissl staining to further investigate whether FUNDC1 plays a protective role against SCI. There were significantly fewer spinal cord anterior horn neurons in the SCI group compared with the sham group. FUNDC1 overexpression significantly increased the number of surviving neurons (Fig. 1E, F).

These results suggest that early activation of FUNDC1 protects against SCI and promotes recovery from SCI. However, the mechanism behind the protective effect of FUNDC1 on SCI needs to be further investigated.

### FUNDC1 overexpression enhanced autophagy and inhibited apoptosis after SCI

**Fig. 2:**
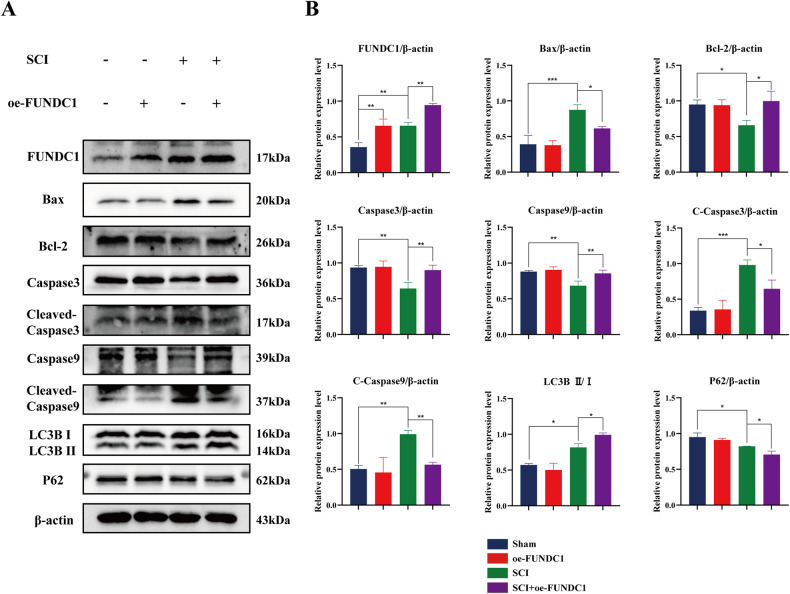
FUNDC1 overexpression enhanced autophagy and inhibited mitochondria-mediated apoptosis in rat spinal cord neurons. **A** Classical schematic of western blotting for FUNDC1, apoptosis- and autophagy-related proteins after SCI and/or FUNDC1 overexpression. **B** Semi-quantitative analysis of FUNDC1, apoptosis, and autophagy-related protein expression (mean ± SD, *n* = 3). Two-way ANOVA was used to compare the groups. ***p < 0.001, **p < 0.01, and *p < 0.05.

FUNDC1, as a hypoxia-induced mitophagy receptor, may be closely related to autophagy. Therefore, we measured the expression of autophagy-specific marker proteins, LC3BII and LC3BI, and autophagic flow-associated protein P62 by western blotting. It was found that the LC3B II/I ratio was increased after SCI, and P62 was downregulated. FUNDC1 overexpression exacerbated these changes. These findings suggest that SCI induces cellular autophagy, and FUNDC1 overexpression further enhances cellular autophagy (Fig. 2A, B).

Apoptosis is an important mode of neuronal death in SCI. To investigate the effect of FUNDC1 on apoptosis, we also examined the expression levels of Bax, Bcl-2, caspase3, cleaved-caspase3, caspase9, and cleaved-caspase9 by western blotting. We found that the expression of the pro-apoptotic proteins Bax, cleaved-caspase3, and cleaved-caspase9 was upregulated in the SCI group compared with the sham group. The expression of the anti-apoptotic protein Bcl-2, as well as caspase-3 and caspase9, was downregulated; however, FUNDC1 overexpression significantly reversed these changes. These results suggest that FUNDC1 may protect neurons by inhibiting apoptosis after SCI (Fig. 2A, B).

### FUNDC1 overexpression enhanced neuronal activity in vitro

**Fig. 3:**
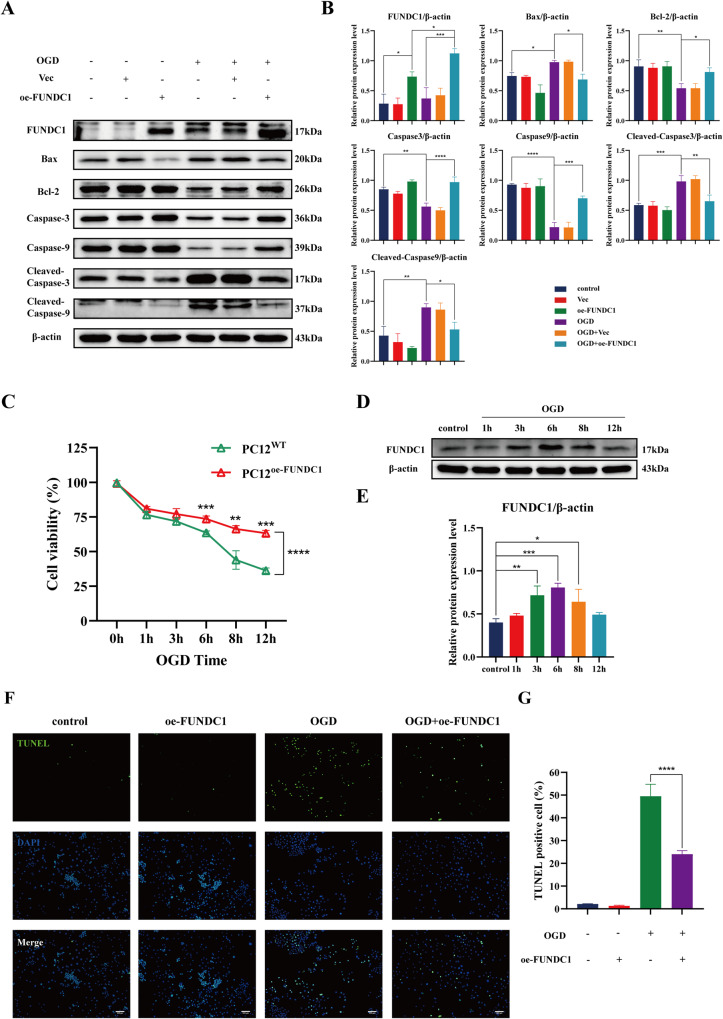
FUNDC1 inhibited apoptosis and enhanced the function of PC12 cells after OGD. **A**, **B** Western blotting was used to analyze the expression levels of FUNDC1, Bax, Bcl2, caspase3, caspase9, cleaved-caspase3, and cleaved-caspase9 (mean ± SD, *n* = 3). **C** Cell viability of PC12^WT^ and PC12^oe-FUNDC1^ cells after different durations of OGD (mean ± SD, *n* = 5). **D**, **E** FUNDC1 protein expression levels in PC12 after different durations of OGD (mean ± SD, *n* = 3). **F**, **G** TUNEL assay was used to detect the apoptosis rate in each group of PC12 cells (mean ± SD, *n* = 3). One-way or Two-way ANOVA was used to compare the groups. ****p < 0.0001, ***p < 0.001, **p < 0.01, and *p < 0.05.

Oxygen-glucose deprivation (OGD) model is a relatively mature ischemia model in vitro. It can better mimic the extracellular environment in the early stage of SCI [22, 23]. Therefore, we utilized an in vitro OGD model to simulate SCI and investigate the protective effects of FUNDC1 with their underlying mechanisms on SCI. First, we used lentiviral transfection of PC12 cells to construct a stable transgenic strain with FUNDC1 gene overexpression (PC12^oe-FUNDC1^/oe-FUNDC1). The overexpression efficiency was detected by western blotting. The results showed that FUNDC1 expression was significantly higher in the oe-FUNDC1 group than in the control group and plasmid null group (Vec group) (Fig. 3A, B).

NGF was used to induce the differentiation of PC12 cells. We subsequently examined the effect of different durations of OGD on cell activity. The survival rate of PC12^oe-FUNDC1^ cells was higher than that of control PC12 cells at all OGD time points, especially after 6 h of OGD (Fig. 3C). In addition, we also measured FUNDC1 expression in PC12 cells after different durations of OGD. It was found that with the prolongation of OGD, FUNDC1 expression gradually increased and then decreased, reaching its peak after 6 h (Fig. 3D, E).

### FUNDC1 attenuated OGD injury by inhibiting mitochondrial apoptotic pathway in vitro

We used OGD 6 h as the time point of OGD injury in the subsequent study in vitro. First, we measured the apoptosis rate by TUNEL staining. The results showed that the proportion of TUNEL-positive cells was higher in the OGD group compared with the control group. The percentage of TUNEL-positive cells was higher in the OGD+oe-FUNDC1 group compared with the control group, but it was significantly lower compared with the OGD group (Fig. 3F, G). In addition, western blotting showed that OGD enhanced the expression of Bax, cleaved-caspase3, and cleaved-caspase9 and decreased the expression of Bcl-2, caspase-3, and caspase9. However, FUNDC1 overexpression significantly reversed these changes (Fig. 3A, B).

To further validate FUNDC1’s role in apoptosis, we knocked down FUNDC1 in PC12 cells using lentiviral transduction (shFUNDC1). Western blots were performed to confirm protein loss. FUNDC1 protein expression decreased within the shFUNDC1 cells compared to the control groups. Following FUNDC1 knockdown in the OGD model, Western blotting also revealed that the levels of Bax, cleaved caspase3, and cleaved caspase9 markedly increased, while Bcl-2 decreased significantly (Fig. S1).

Altogether, these results indicate that FUNDC1 can reduce OGD-induced mitochondria-dependent apoptosis.

### FUNDC1 exerted a cytoprotective effect by reducing OGD-induced mitochondrial damage in vitro

**Fig. 4:**
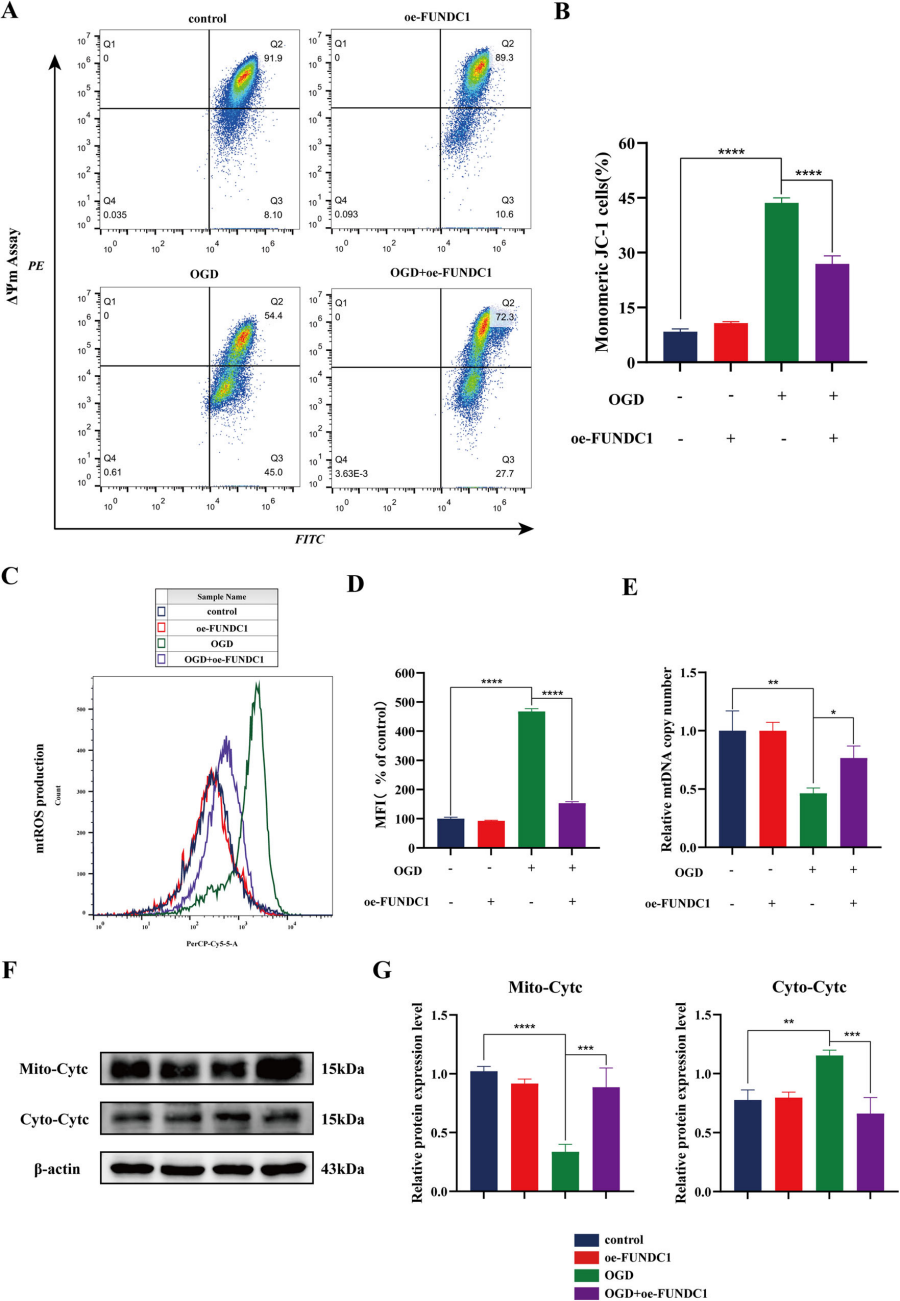
FUNDC1 protected mitochondria against OGD in vitro. **A** Mitochondrial membrane potential was detected using JC-1 staining to assess mitochondrial function. In each graph, region Q3 shows cells containing JC-1 monomer, representing impaired mitochondria, while region Q2 shows cells containing JC-1 polymer, representing normal mitochondria. **B** JC-1 staining was quantified to show the percentage of cells with monomeric JC-1 in each group (mean ± SD, *n* = 3). **C**, **D** Flow cytometry was used to detect mtROS production in each group of cells (mean ± SD, *n* = 3). **E** The relative mtDNA copy number was determined by qPCR with primers for *mt-CO1* and *β-actin*. Data was normalized to *β-actin* gene and then normalized to control (mean ± SD, *n* = 3). **F**, **G** Western blotting for measuring CytC expression in the mitochondria and cytoplasm (mean ± SD, *n* = 3). Two-way ANOVA was used to compare the groups. ***p < 0.001, **p < 0.01, and *p < 0.05.

Spinal cord neurons are susceptible to ischemic injury due to their high energy demand. Mitochondria are the main target of ischemic injury and play an important role in the pathogenesis of SCI [24]. Therefore, we further analyzed whether FUNDC1 can improve mitochondrial function in addition to inhibiting the mitochondrial apoptosis pathway. We assessed mitochondrial membrane potential by JC-1 staining. OGD reduced mitochondrial membrane potential, while FUNDC1 overexpression significantly reversed these changes (Fig. 4A, B). Then, the ROS produced in the mitochondria (mtROS) release assay revealed that OGD increased mtROS production, while FUNDC1 overexpression hindered mtROS overproduction (Fig. 4C, D). In addition, relative quantification of mtDNA copy number was performed by the ratio of the copy number of *mt-CO1* to the copy number of *β-Actin*. The results showed that OGD resulted in a significant reduction in cellular mtDNA copy number, and OGD-mediated loss of mtDNA was limited by FUNDC1 overexpression (Fig. 4E).

Mitochondrial damage can increase mitochondrial permeability, allowing CytC leakage from the mitochondria into the cytoplasm, thereby activating caspase9 [25]. Thus, CytC release is both a marker of mitochondrial damage and an early sign of mitochondria-dependent apoptosis. Western blotting indicated that CytC expression was increased in the cytoplasm and decreased in the mitochondria after OGD. OGD resulted in CytC leakage from the mitochondria to the cytoplasm. However, FUNDC1 overexpression significantly decreased CytC release (Fig. 4F, G).

Taken together, these results consistently demonstrate that FUNDC1 overexpression limits OGD-induced mitochondrial damage in vitro, thereby exerting a cytoprotective effect.

### FUNDC1 modulated mitochondrial function and apoptosis by mediating mitophagy

Mitophagy is a selective form of autophagy targeting the damaged mitochondria. It is critical for maintaining mitochondrial function and reducing apoptosis during stress conditions. We investigated whether the neuroprotective effect of FUNDC1 is related to mitophagy.

**Fig. 5:**
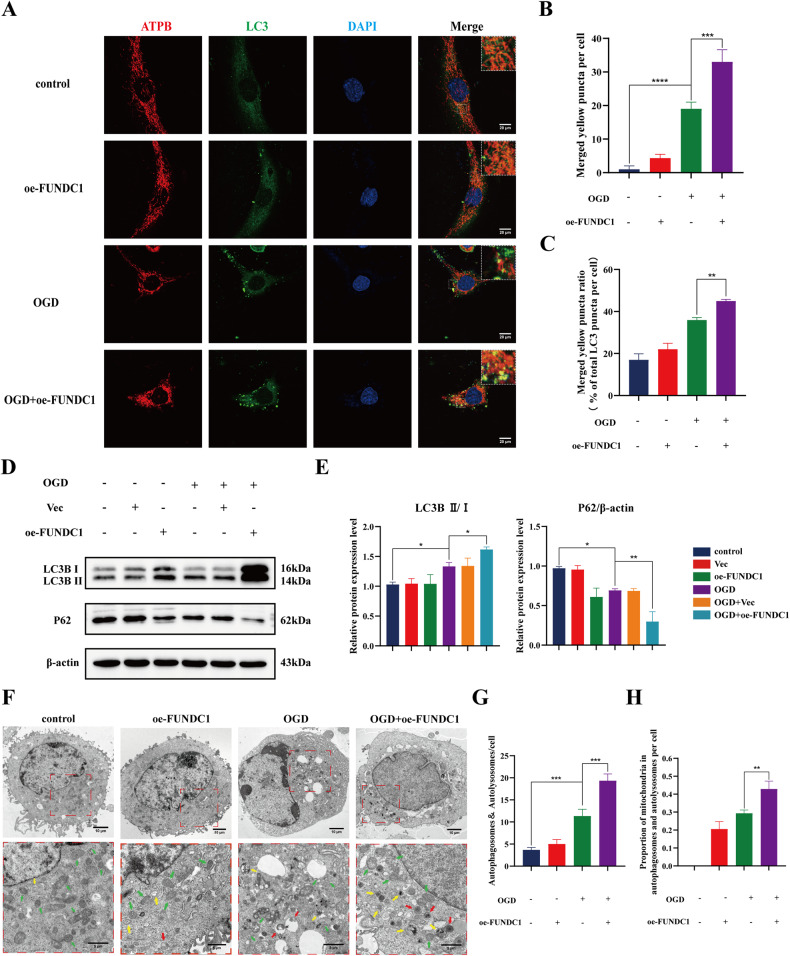
FUNDC1 enhanced mitophagy in OGD PC12 cells. **A**–**C** Co-localization of ATPB with LC3 was observed using laser confocal microscopy to assess mitochondrial autophagy. ATPB was labeled with red fluorescence, LC3 was labeled with green fluorescence, and yellow fluorescence represents the co-localization of ATPB with LC3. All cell nuclei were stained using DAPI (blue). The yellow puncta in each group of cells was quantified (mean ± SD, *n* = 3). Scale bar = 20 μm. **D**, **E** Western blotting analysis of the expression of LC3B and P62 (mean ± SD, *n* = 3). **F**–**H** TEM was used to analyze cellular autophagy. The boxed regions in the upper panels are enlarged in the lower panels. Green arrows: mitochondria; yellow arrows: autophagy; red arrows: mitophagy. Autophagosomes and autophagolysosomes as well as mitochondria in the autophagosomes and autophagolysosomes in each group of cells was quantified (mean ± SD, *n* = 3). Scale bar = 10 μm or 5 μm. Two-way ANOVA was used to compare the groups. ****p < 0.0001, ***p < 0.001, **p < 0.01, and *p < 0.05.

We performed a series of in vitro experiments to further confirm the effect of FUNDC1 on mitophagy. LC3 is a structural protein of autophagosome. Immunofluorescence co-localization of the mitochondrial marker protein ATPB with LC3 was used to observe the changes in mitophagy. The results showed that the green fluorescence of LC3 was enhanced and aggregated, and the number of yellow puncta and aggregates increased after OGD, which indicated that OGD increased the expression of LC3 and the co-localization of LC3 with mitochondria. FUNDC1 overexpression significantly enhanced these alterations (Fig. 5A, B). Meanwhile, compared to the OGD group, the ratio of yellow puncta to the total LC3 puncta per cell was significantly increased in the cells of the OGD+oe-FUNDC1 group (Fig. 5C). In addition, western blotting indicated similar results. OGD increased LC3B II/I ratio and decreased P62 expression. FUNDC1 overexpression significantly enhanced these alterations compared with the OGD group (Fig. 5D, E).

We also used TEM mainly to analyze the level of autophagy in each group of cells by observing the number of autophagosomes (including mitochondrial autophagosomes) and autophagolysosomes. The results showed a significant increase in autophagosomes as well as autophagolysosomes in the OGD group compared to the control group. FUNDC1 overexpression significantly increased these alterations. Also, there was not only autophagy (yellow arrow), but also more mitophagy (red arrow) in the OGD+oe-FUNDC1 group (Fig. 5F, G). Compared with the OGD group, the proportion of mitochondria in autophagosomes and autolysosomes was significantly increased in the cells of the OGD+oe-FUNDC1 group (Fig. 5H). These results suggest that OGD enhances mitophagy, and FUNDC1 overexpression can further enhance mitophagy.

**Fig. 6:**
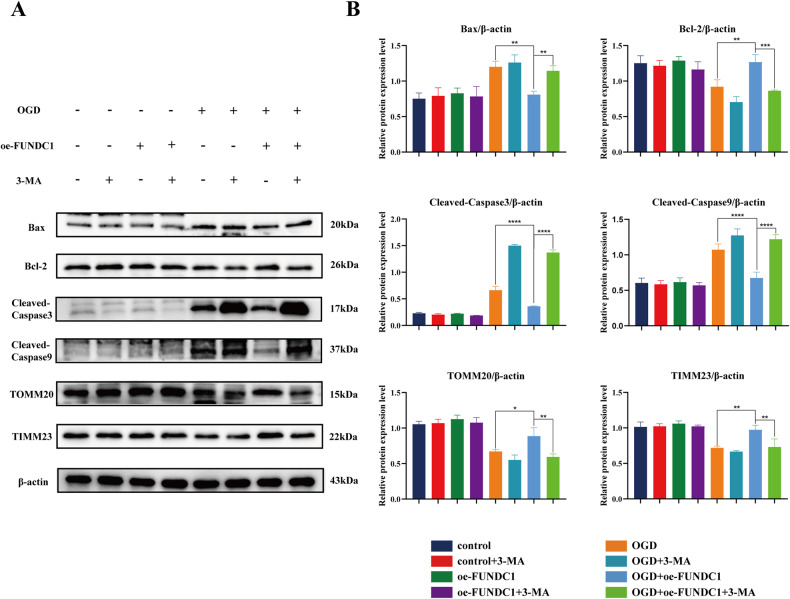
3-MA attenuated the protective effects of FUNDC1 against apoptosis and mitochondrial dysfunction by inhibiting autophagy. **A** Classical schematic of western blotting for mitochondria-specific proteins and apoptosis-related proteins after 3-MA intervention. **B** Semi-quantitative analysis of the expression levels of mitochondria-specific proteins and apoptosis-related proteins (mean ± SD, *n* = 3). Three-way ANOVA was used to compare the groups. ****p < 0.0001, ***p < 0.001, **p < 0.01, and *p < 0.05.

3-methyladenine (3-MA) is widely used as an autophagy inhibitor. It can hinder autophagosome formation by inhibiting type III phosphatidylinositol 3-kinase (PI3K) [26]. To further explore the relationship among FUNDC1, mitochondria, autophagy and apoptosis, 3-MA was used in our study. The results show that FUNDC1 overexpression enhanced the expression of the anti-apoptotic protein Bcl-2 and decreased the expression of the pro-apoptotic proteins Bax, cleaved-caspase-3, and cleaved-caspase-9 after OGD. 3-MA significantly reversed these alterations (Fig. 6A, B). In addition, compared to the OGD+oe-FUNDC1 group, we found that the expression of mitochondrial membrane proteins TOMM20 and TIMM23 significantly decreased after adding 3-MA (Fig. 6A, B).

Taken together, these results suggest that the protective effects of FUNDC1 on mitochondrial dysfunction and apoptosis are mediated by enhancing mitophagy.

## Discussion

SCI is a relatively rare but devastating disease with a heavy economic burden and significant morbidities. According to reports published by the World Health Organization (WHO), the estimated global incidence of SCI is 40 to 80 new cases per million people per year, which means that 250,000 to 500,000 people experience SCI annually. The majority of these cases are traumatic SCI, the main causes of which are car accidents, falls, and violence [27]. Primary injury to the spinal cord can cause a series of pathological changes, which can reduce blood flow to the spinal cord. Disruption of blood flow leads to tissue ischemia and hypoxia, thereby accelerating tissue damage and inducing secondary SCI [28]. Therefore, early intervention for SCI is crucial to stop its progression, prevent secondary injury, and facilitate the repair process. This study investigated the role of FUNDC1 in the early stages of SCI and explored its possible mechanisms. We found that early activation of FUNDC1 protects against SCI and promotes the repair process. In addition, FUNDC1 overexpression enhanced autophagy and reduced mitochondria-dependent apoptosis in the rat model of SCI and in vitro OGD model. Notably, the protective effects of FUNDC1 against apoptosis and mitochondrial dysfunction were mediated through mitophagy.

Mitochondria produce ATP through oxidative phosphorylation, which is necessary for a variety of cellular functions. As mitochondrial metabolism is accompanied by ROS production, the accumulation of damaged mitochondria can activate the mitochondrial apoptotic pathway [29, 30]. The mitochondrial apoptotic pathway is one of the main pathways of apoptosis. Various pro-apoptotic stimuli can decrease mitochondrial membrane potential and increase mitochondrial membrane permeability. These alterations lead to the release of CytC and other pro-apoptotic factors, accelerate apoptotic vesicles formation, and initiate the caspase cascade reaction, eventually leading to apoptosis [29]. SCI often causes mitochondrial damage and is closely associated with apoptosis. Using in vivo and in vitro experiments, Hu et al. [31] reported that compared with the control group, the SCI group had significantly damaged mitochondria. They also reported that after SCI, the number of mitochondria significantly reduced, the crest disappeared, and the mitochondrial vacuolization significantly increased, which were closely related to apoptosis. Using a rat SCI model, Xu et al. [32] also found that mitochondrial damage-related manifestations such as mitochondrial swelling, breakage, and cristae disorder can be observed in pre-apoptotic spinal cord neurons. We observed significant upregulation of pro-apoptotic proteins Bax, cleaved-caspase3, and cleaved-caspase9, and significant downregulation of anti-apoptotic proteins Bcl-2, caspase3, and caspase9 in the SCI models both in vivo and in vitro. In addition, excessive ROS release, CytC leakage, and reduced potential of mitochondrial membrane were observed in vitro. Consistent with previous studies, our findings suggest that SCI can significantly cause mitochondrial damage and activate mitochondria-mediated apoptosis.

Therefore, mitochondrial function must be closely monitored and damaged mitochondria need to be removed in a timely manner. Mitophagy is a selective type of autophagy that can specifically recognize and degrade damaged mitochondria [11]. Mitophagy is closely related to apoptosis. Several studies have reported that mitochondrial apoptosis can be inhibited by early mitophagy to protect cells from ischemic injury. Li et al. [24] found that rapamycin can inhibit neuronal apoptosis after SCI by enhancing mitophagy. Tang et al. [33] also suggested that activation of BNIP3-mediated mitophagy can inhibit apoptosis and protect against renal ischemia-reperfusion injury. Another study reported that hydrogen-rich saline can protect cardiomyocytes against myocardial ischemia-reperfusion-induced inflammatory response and apoptosis through PINK1/Parkin-mediated mitophagy [34]. FUNDC1 is a mitochondrial membrane protein with a classical LIR, closely related to mitophagy [12]. We found that FUNDC1 overexpression can increase LC3II/LC3I ratio, promote LC3 recruitment to the mitochondria, and decrease P62 expression, confirming that FUNDC1 can regulate mitophagy after SCI.

Meanwhile, accumulating evidence shows that FUNDC1, a mitophagy receptor induced by hypoxia, plays a critical role in ischemic injury. Cai et al. [25] found that tPA can improve mitochondrial function and reduce neuronal apoptosis through FUNDC1-mediated mitophagy, thereby protecting neurons against ischemic stroke. Zhou et al. [35] revealed that myocardial ischemia activates FUNDC1-mediated mitophagy, which reduces cardiomyocyte apoptosis. Furthermore, they found that Ripk3 upregulation facilitates mitochondria-mediated apoptosis by inhibiting FUNDC1-dependent mitophagy. Zhang et al. [36] also observed that mitophagy receptor FUNDC1 could regulate mitochondrial homeostasis and protect the heart from ischemia-reperfusion injury. Similarly, we found that FUNDC1 overexpression not only enhanced mitophagy but also inhibited the mitochondrial pathway of apoptosis and improved mitochondrial function, thereby protecting spinal cord neurons from ischemic injury. In addition, the autophagy inhibitor, 3-MA, attenuated the protective effects of FUNDC1 against apoptosis and mitochondrial dysfunction. These findings confirm that the protective effects of FUNDC1 against apoptosis and mitochondrial dysfunction are mediated through mitophagy.

For the first time, this study demonstrated that FUNDC1 can enhance mitophagy, reduce early apoptosis, and improve mitochondrial function after SCI. However, this study has some limitations. First, the relevant upstream and downstream signaling molecules, as well as the signaling pathways involved in FUNDC1-mediated mitophagy, were not explored in depth. Second, instead of using spinal cord primary neurons for in vitro experiments, we used NGF-treated PC12 cells.

## Conclusion

In conclusion, the results of this study suggest that early activation of FUNDC1 can protect against SCI and promote the repair process by mediating mitophagy, inhibiting the mitochondrial apoptotic pathway, and improving mitochondrial function. These results indicate that FUNDC1 can be used as a target for the early treatment of SCI.

## Materials and methods

### Animals and experimental groups

Adult Sprague-Dawley rats, weighing 220–250 g, were used in this experiment and were purchased from the Animal Experiment Center of Fujian Medical University. All experiments were approved by the Animal Ethics Committee of Fujian Medical University and conducted in accordance with the National Institutes of Health Guide for the Care and Use of Laboratory Animals. All rats were kept in a room with 22-24 °C temperature, 45–55% humidity, and 12-hour light-dark cycles. The animals had free access to food and water. Thirty-six rats were randomly divided into 4 groups, including the sham group (*n* = 9), AAV-Fundc1 group (*n* = 9), SCI group (*n* = 9), and SCI + AAV-Fundc1 group (*n* = 9) to investigate the transfection efficiency of AAV-Fundc1 and the effect of FUNDC1 overexpression on SCI.

### Construction and delivery of AAV viral vectors

The AAV-Fundc1 virus used in this experiment was purchased from GEMA Genetics. We selected AAV-DJ, which is suitable for nervous system transfection, to overexpress FUNDC1. The empty AAV virus (AAV-NC) was used as a control. Local intramedullary injection of the AAV virus was performed by a Hamilton microinjector and locator (RWD Life Science Corp, 68097). Briefly, we first anesthetized the rats using an intraperitoneal injection of 1% pentobarbital (50 mg/kg). Then, we performed a laminectomy at T10 to expose the spinal cord. A Hamilton microinjector was obliquely inserted into the spinal cord at a distance of approximately 1.0 mm lateral to the midline of the spinal cord (penetration depth of approximately 2 mm). The Hamilton microinjector was fixed and 5 μL of AAV virus was injected (4.79 × 1012 V.G./ml) into the left and right spinal cord at a rate of 1 μL/min. After injection, the needle was lagged for 5 min and slowly withdrawn. After adequate hemostasis, the wound was closed with layer-by-layer sutures. For the sham group, rats underwent the same surgery except for spinal cord contusion. After surgery, rats intraperitoneally received cefmetazole (100 mg/kg/day for 3 days). Injured rats were assisted to urinate twice daily until urinary retention disappeared.

### Construction of SCI model in rats

The SCI model was established in rats according to a modified Allen’s method. One week after virus injection, rats were anesthetized with the intraperitoneal injection of 1% pentobarbital (50 mg/kg). The T10 vertebral plate was removed to expose the spinal cord, and the rats were immobilized on a modified Allen’s impinger (RWD Life Science Corp, 68097). The T10 spinal cord segment of the rat was accurately positioned for impact. The impact head had a diameter of 2.5 mm and weighed 15 g. It was released 10 cm above the surgical site and then left to rest for 3 min. Tail spasm and bilateral hindlimb paralysis after the impact were assumed as signs of successful modeling.

### Assessment of locomotor capacity

The exercise capacity assessment used the 21-point (0-21) BBB scale to assess the rats’ behaviors before and 24 h, 48 h, 72 h, 5 days and 7 days by two blinded investigators following surgery. Assessments were based on observations of the rats’ unrestricted hindlimb movement in a field at a certain time every morning of the testing period, during which rats were able to walk without restrictions on a field for 5 min. In addition, BBB score was performed immediately after the rats regained consciousness. Rats with scores higher than 5 were excluded because they failed in modeling, and backup rats were used for modeling supplement.

Seven days following surgery, an assessment of the rats’gait and motor coordination was conducted. Their paws were covered with differently colored dye. They were put on permeable paper in a cage and encouraged to walk directly forward. Their footprint pattern was digitized and an overall image was applied to determine coordination.

### Spinal cord tissue processing and H&E and Nissl staining

Rats were anesthetized one week after SCI, perfused with 0.9% NaCl transcardiac, and then perfused with 0.1 M phosphate buffer containing 4% paraformaldehyde (PFA). The spinal cord was entirely removed immediately after perfusion. It was fixed in 4% PFA overnight before paraffin embedding. Paraffin sections (4 μm thick) of spinal cord tissue were made on poly-l-lysine-coated slides. H&E and Nissl staining were performed after dewaxing for histopathological examination. Images were taken using a light microscope (Leica, DM2500). ImageJ software (NIH) was used to analyze the number of surviving neurons.

### Cell culture and OGD model construction

Nerve growth factor (NGF) can induce rat pheochromocytoma cells (PC12 cells) to differentiate into a morphology similar to that of primary and mature neurons [37, 38]. The PC12 cells used in this study were purchased from the cell bank of the Chinese Academy of Sciences (Shanghai, China) and cultured with the Roswell Park Memorial Institute 1640 medium (Gibco) containing 10% horse serum (Gibco), 5% fetal bovine serum (Gibco), and 1% penicillin/streptomycin (Gibco) at 37°C in a humidified culture incubator with 5% CO_2_.

PC12 cells were inoculated at a density of 1 × 10^6^ cells per dish in poly-L-lysine-coated 6-cm culture dishes. After adhering, the original medium was replaced with RPMI-1640 induction medium containing 50 ng/mL NGF, 10% FBS, and 1% penicillin/streptomycin. One time per 2 days, the medium was switched until the neurite length was greater than the cell body of the individual cells at the 5th day. After differentiation, cells were treated.

The oxygen-glucose deprivation (OGD) model is a well-established in vitro ischemic model. We used NGF-induced PC12 cells to construct an OGD model to simulate spinal cord ischemic injury in vitro. The OGD model was established by subjecting these cells to serum-free Hanks’ balanced salt solution (Gibco) and a hypoxic environment (1% O_2_, 5% CO_2_, and 94% N_2_) for 6 h.

### Overexpression and knockdown of FUNDC1 gene in PC12 cells

For upregulating FUNDC1 expression in PC12 cells, we obtained a full-length FUNDC1 sequence fragment using a PCR method and subsequently cloned it into vector LV18, verified by DNA sequencing and transfected 293 T cells. The collected lentivirus was used to infect PC12 cells and the cells were screened with a cell culture containing 5ug/mL puromycin. Finally, PC12 cells were collected for western blotting to assess the efficiency of FUNDC1 overexpression.

To knockdown the expression of FUNDC1 protein, a FUNDC1 shRNA was developed to silence the FUNDC1 genes. FUNDC1 shRNA oligonucleotides with the LV2N (U6/Puro) vector was used to construct the Lenti-FUNDC1 shRNA vectors. Lentiviruses (LentiFUNDC1 shRNA or Lenti-shRNA) (MOI = 20) were applied for transfecting PC12 cells with 5 μg/ml polybrene for 24 h based on the manufacturer’s directions (GenePharma, Shanghai, China). After 5 μg/ml puromycin selection, the PC12 cells were harvested for western blot to evaluate FUNDC1 knockdown efficiency.

### Cell viability assay

The activity of PC12 cells under different durations of OGD was detected using the CCK-8 method. Briefly, six 96-well plates were grouped according to different durations of OGD injury (1 h, 3 h, 6 h, 8 h, and 12 h), with normal cells as the control group, and five replicate wells were used in each group. Cells were inoculated uniformly in 96-well plates at a density of 10,000 cells/well. After adhereing, the medium was removed, washed twice with PBS, and cells in each well underwent OGD after adding 200 μL of HBSS serum-free medium. Each group of cells was taken out simultaneously at the end of modeling, the medium was removed, and washed twice with PBS. Then, 100 μL of HBSS serum-free medium containing 10% CCK-8 was re-added. They were incubated for 1.5 h in a 37 °C incubator. Finally, the absorbance was read at 450 and 630 nm using a microplate reader (BioTek, EPOCH).

### Western blotting

Total proteins were extracted from cells and tissues with RIPA reagent supplemented with phenylmethylsulfonyl fluoride (PMSF) and phosphatase inhibitor. Mitochondrial and cytoplasmic proteins were extracted with a commercial mitochondrial isolation kit (Thermo Fisher). Protein concentrations were determined using a BCA kit (Boost). The proteins were transferred to PVDF membranes by sodium dodecyl sulfate-polyacrylamide gel electrophoresis. Afterward, PVDF membranes were blocked with 5% skimmed milk for 2.5 h at room temperature, followed by overnight incubation with primary antibodies at 4 °C. Then, the PVDF membranes were incubated with horseradish peroxidase-labeled secondary antibodies (1:5000, Dingguo) for 1.5 h at room temperature. The chemiluminescence agent (Beyotime Biotech) was used to show immunolabeled bands. Protein expression levels were determined using ImageJ software (NIH) based on densitometry. The following primary antibodies were used in this study: anti-FUNDC1 (1:1000, Abcam, ab74834), anti-Bax (1:1000, CST, #5023), anti-Bcl2 (1:1000, Abcam, ab692), anti-Caspase3 (1:1000, CST, #9662), anti-Cleaved-Caspase3 (1:1000, CST, #9664), anti-Caspase9 (1:1000, CST, #9508), anti-Cleaved-Caspase9 (1:1000, CST, #20750), anti-LC3B (1:1000, Abcam, ab192890), anti-p62 (1:1000, CST, #8025), anti-CytC (1:1000, ProteinTech, 10993-1-AP), anti-TOMM20 (1:1000, Abcam, ab56783), anti-TIMM23(1:1000, Santa Cruz Biotechnology, sc-514463), anti-ATPB (1:1000, Abcam, ab14730), anti-β-actin(1:1000, CST, #3700).

### TUNEL assay

After fixation and permeabilization of PC12 cells, TUNEL labeling working solution (Elabscience) was added and dark incubated for 1 h. The nuclei were stained with DAPI and the number of TUNEL-positive cells was quantified using a fluorescent microscope (Leica, DMi8).

### Detection of mitochondrial membrane potential (ΔΨm)

The JC-1 assay kit (MedChemExpress) was used for detecting mitochondrial membrane potential. According to the protocol, we collected cells in centrifuge tubes using EDTA-free trypsin. Thereafter, cell culture medium containing JC-1 staining solution was added. After mixing, the cells were incubated in a 37 °C incubator for 20 min. Cells were mixed every 5 min to allow sufficient exposure to the probe. After incubation, the cells were centrifuged at 400 × *g* for 3 min at 4 °C to obtain the precipitate. Then, the precipitate was washed twice with PBS, and flow cytometry (BD bioscience, FACSCalibur) was used for the assay.

### ROS produced in the mitochondria detection

The MitoSOX Red mitochondrial superoxide indicator (MedChemExpress) was used for detecting mtROS. According to the protocol, MitoSOX Red was first diluted with serum-free culture medium at 1:1000 to a final concentration of 5 μmol/L. Cells were collected and suspended in diluted MitoSOX Red and incubated in a 37°C cell incubator for 20 min. They were mixed every 5 minutes to allow complete exposure to the probe. The cells were then centrifuged at 800 rpm for 3 min to obtain the precipitate. The cells were washed twice with a serum-free cell culture medium. Finally, flow cytometry (BD bioscience, FACSCalibur) was used for the assay.

### Mitochondrial DNA content determination

Relative quantification of cellular mtDNA copy number was performed by the ratio of the copy number of the mitochondrially encoded *cytochromec oxidase I* (*mt-CO1*) gene sequence in mtDNA to the copy number of the housekeeping gene *β-Actin* sequence. Total DNA (mtDNA and nuclear genomic DNA) was extracted using the Wizard@Genomic DNA Purification Kit (Promega). The relative mtDNA copy number was determined by qPCR with primers for the mt-CO1 gene and the housekeeper gene. PCR amplification was performed with real-time PCR using SYBR Green qPCR Master Mix (MCE) in Applied Biosystems real-time PCR system (ABI, 7500). The relative changes for each sample were determined by normalizing to the housekeeper gene levels.

Primers used were:

*mt-CO1*, forward, 5ʹ-GGAGCAGTATTCGCCATCATAG-3ʹ;

*mt-CO1*, reverse, 5ʹ-TGTGGTGTAAGCATCTGGATAATC-3ʹ;

*β-Actin*, forward, 5ʹ-CGCGAGTACAACCTTCTTGC -3ʹ;

*β-Actin*, reverse, 5ʹ-CCTTCTGACCCATACCCACC-3ʹ;

### Immunofluorescence microscopy

PC12 cells were fixed in 4% paraformaldehyde. Then, PC12 cells were treated with 0.2% Triton X-100 (Solarbio) to make them permeable. Thereafter, they were blocked with diluted 5% BSA (Sigma) for 30 min. Subsequently, the cells were incubated with the primary antibodies overnight at 4 °C. After washing with PBS, the cells were dark incubated with the fluorescent secondary antibody for 1 h at room temperature. Then, the cell nuclei were re-stained with DAPI for 15 min. Finally, a confocal fluorescence microscope (Leica, TCS SP8) was used to obtain fluorescence images.

### Transmission electron microscope

PC12 cells were collected and fixed with pre-chilled 2% glutaraldehyde solution at 4 °C for 2 h. Then, the cells were stained with 2% uranyl acetate solution for 2 h and dehydrated in 50%, 70%, 90%, and 100% acetone. Finally, cells were embedded and prepared into ultrathin sections for measurement by an electron microscope (Hitachi, HT7700).

### Statistical analysis

All data are expressed as mean ± standard deviation. Data from at least three independent replicates were analyzed using SPSS software (version 25.0) and Prism v8.0 software (GraphPad). Differences between multiple groups were assessed using ANOVA, followed by Tukey’s multiple comparison test. *p* < 0.05 was considered statistically significant.

### Supplementary information

SUPPLEMENTAL MATERIAL

Original Data File

## Data Availability

The datasets used and/or analyzed during the current study are available from the corresponding author on reasonable request.
