# Supplementary material for: FUNDC1-induced mitophagy protects spinal cord neurons against ischemic injury
Source: Cell Death Discov. 2024 Jan 5;10:4. doi: 10.1038/s41420-023-01780-9 (PMC10766648; doi:10.1038/s41420-023-01780-9)
Supplement: Supplementary file 1 — SUPPLEMENTAL MATERIAL [file 41420_2023_1780_MOESM1_ESM.docx]

# Supporting information

**FUNDC1-Induced Mitophagy Protects Spinal Cord Neurons against Ischemic Injury**

Dehui Chen, MD^1†^. Linquan Zhou, MD^1†^. Gang Chen, MD^1†^. Taotao Lin, MD^1^. Jiemin Lin, MM^2^. Xin Zhao, MM^3^. Wenwen Li, MM^1^. Shengyu Guo, MM^1^. Rongcan Wu, MM^1^. Zhenyu Wang, MD, PhD^1*^. Wenge Liu, MD, PhD^1*^.

^1^Department of Orthopedics, Fujian Medical University Union Hospital, Fuzhou 350001, Fujian, China.

^2^Department of Rehabilitation Medicine, The First Affiliated Hospital of Fujian Medical University, Fuzhou 350001, Fujian, China.

^3^Department of Rehabilitation Therapy, Jiangsu Rongjun Hospital, Wuxi 214035, Jiangsu, China.

^†^Dehui Chen, Linquan Zhou and Gang Chen contributed equally to this work.

**^*^**Corresponding author:

Wenge Liu (wengeunion@fjmu.edu.cn)

Zhenyu Wang (zhenyu_wang@fjmu.edu.cn.)


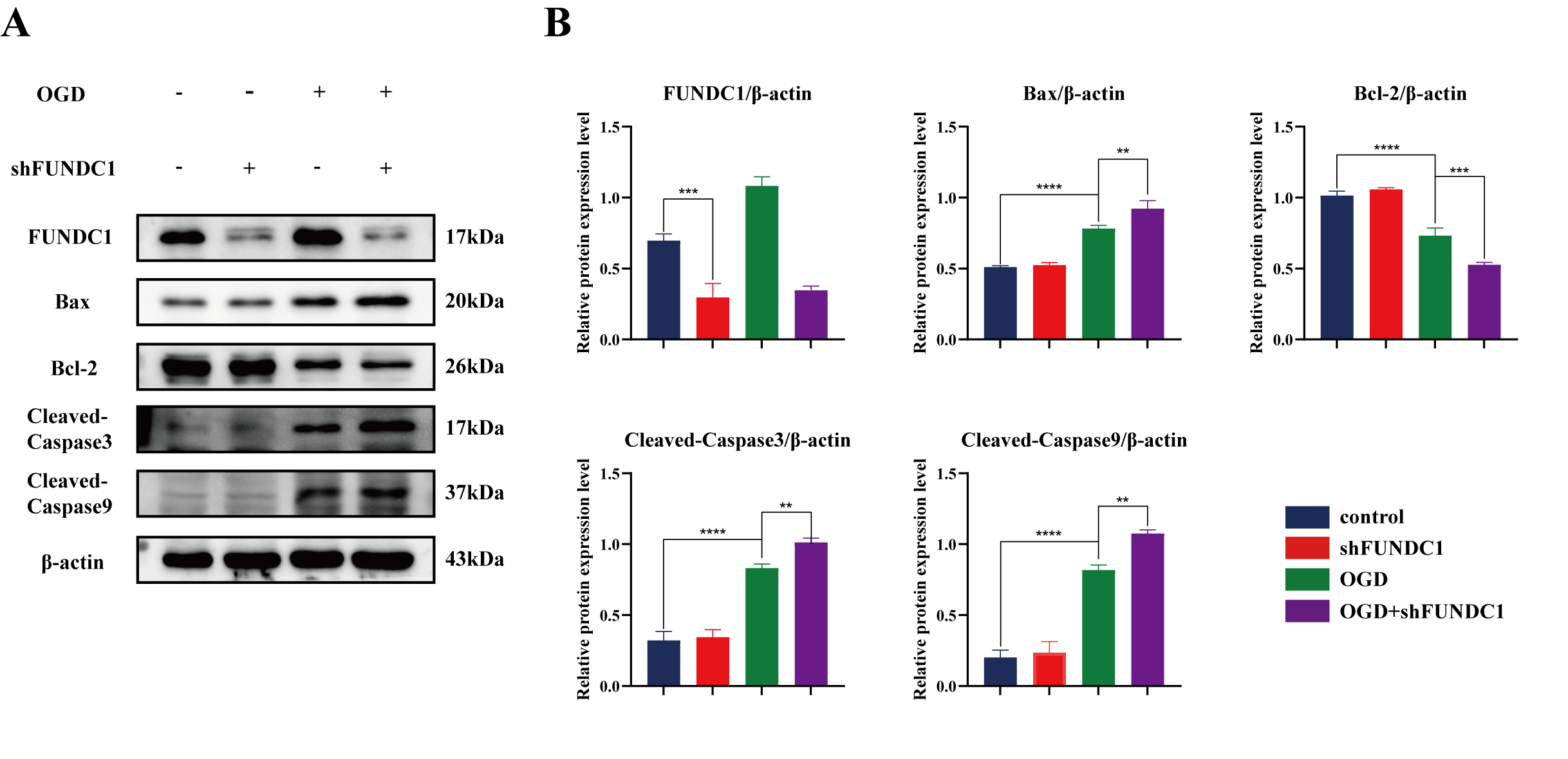


**Fig. S1. FUNDC1 knockdown upregulated mitochondria-mediated apoptosis in OGD PC12 cells. (A)** Classical schematic of western blotting for FUNDC1, Bax, Bcl2, cleaved-caspase3, and cleaved-caspase9 after OGD and/or FUNDC1 knockdown. **(B)** Semi-quantitative analysis of the expression levels of FUNDC1, Bax, Bcl2, cleaved-caspase3, and cleaved-caspase9 (mean ± SD, n = 3). Two-way ANOVA was used to compare the groups. ****p<0.0001, ***p<0.001, and **p<0.01.
