# Supplementary material for: FUNDC1-induced mitophagy protects spinal cord neurons against ischemic injury
Source: Cell Death Discov. 2024 Jan 5;10:4. doi: 10.1038/s41420-023-01780-9 (PMC10766648; doi:10.1038/s41420-023-01780-9)

Figure 2A

FUNDC1:

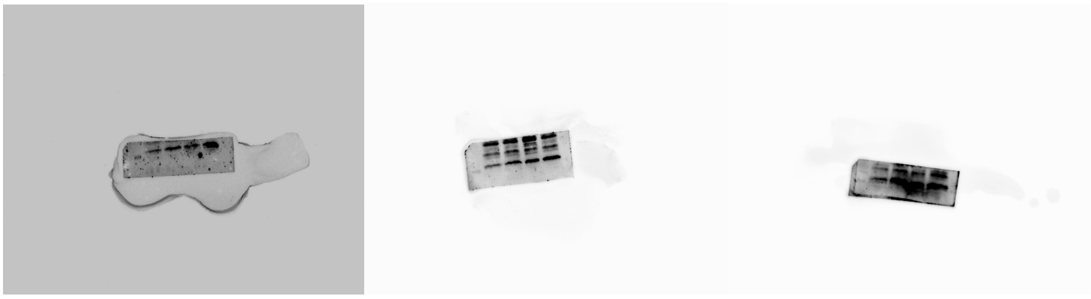

Bax:

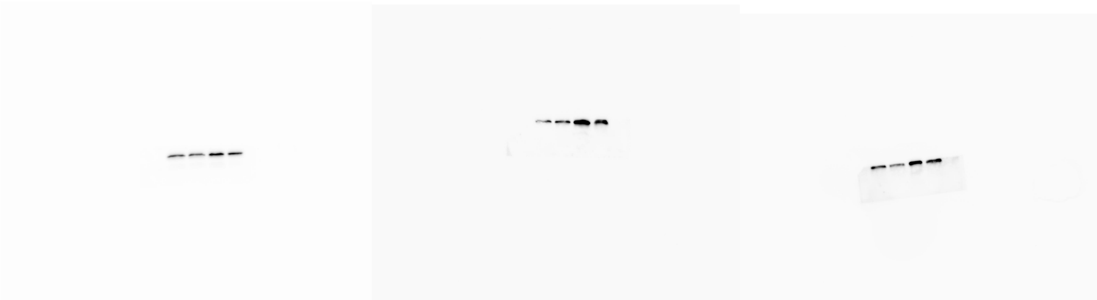

Bcl-2:

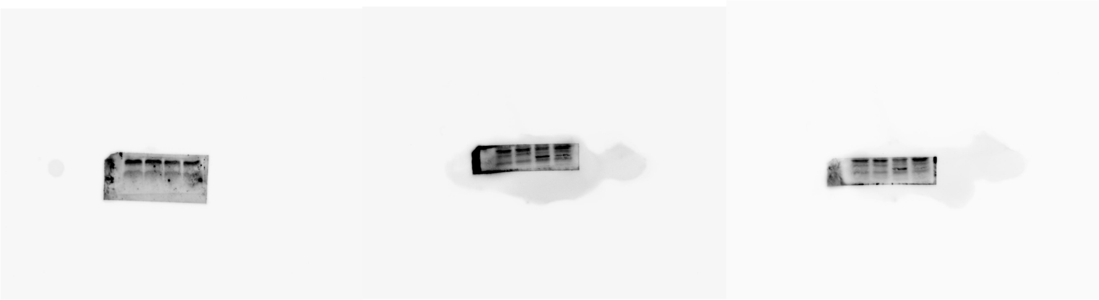

Caspase3:

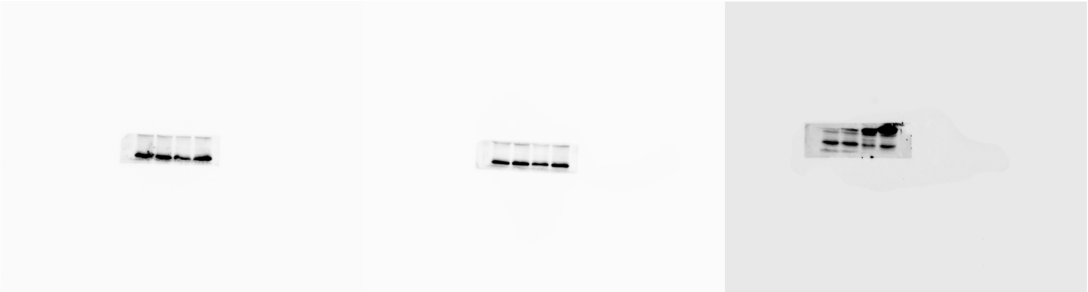

Cleaved-Caspase3:

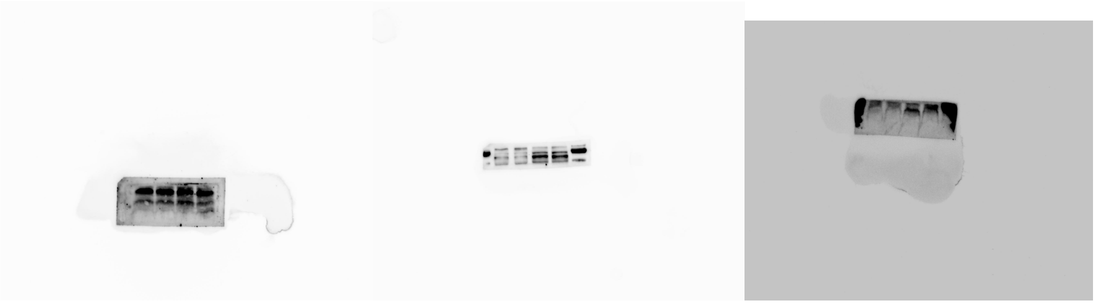

Caspase9:

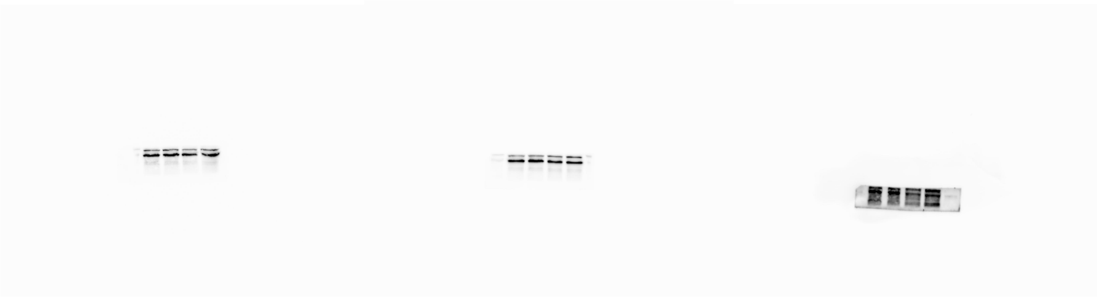

Cleaved-Caspase9:

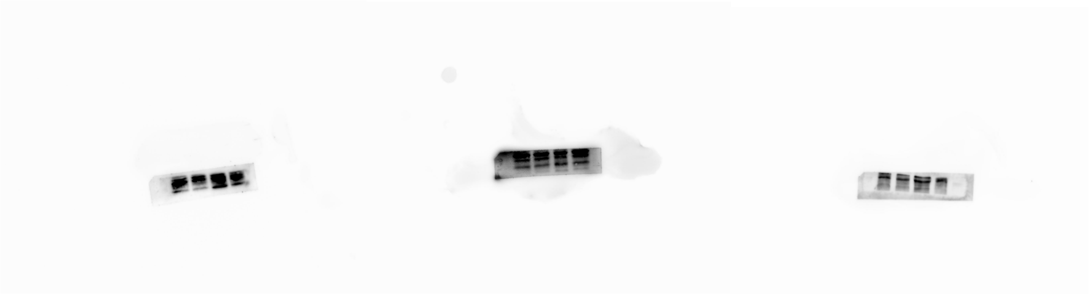

LC3B:

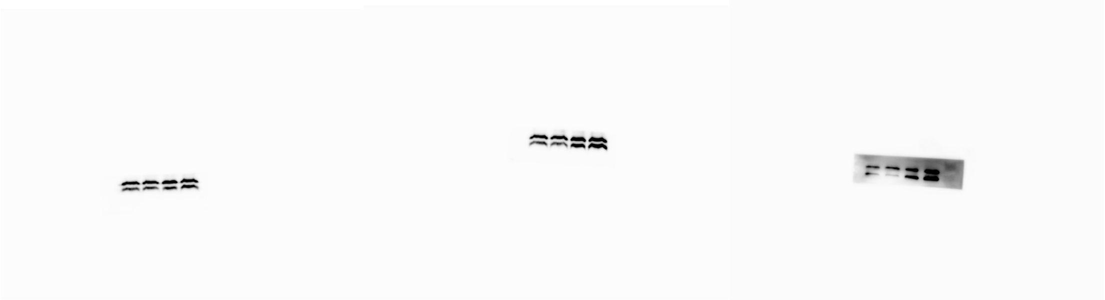

P62:

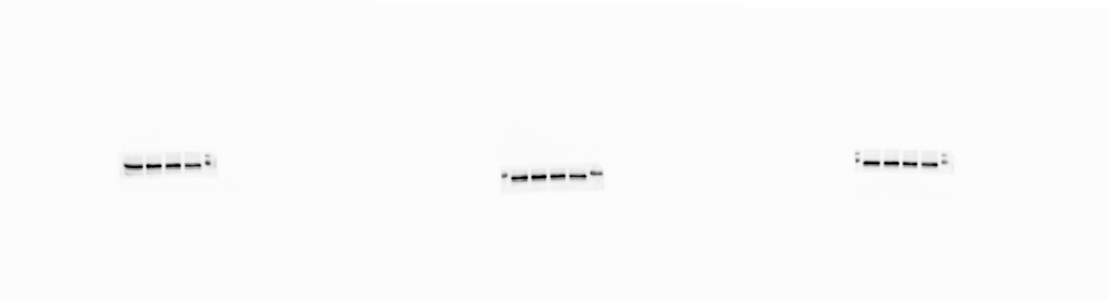

Figure 3A

FUNDC1:

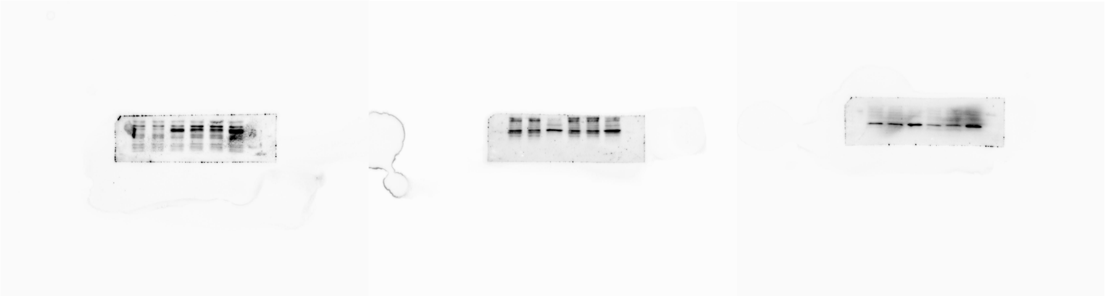

Bax:

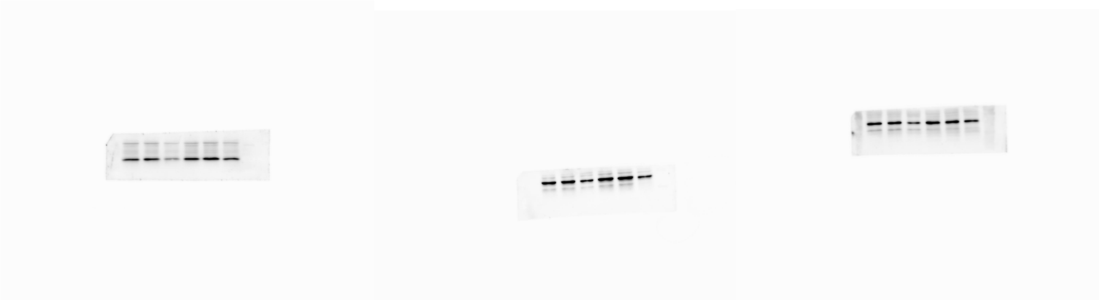

Bcl-2:

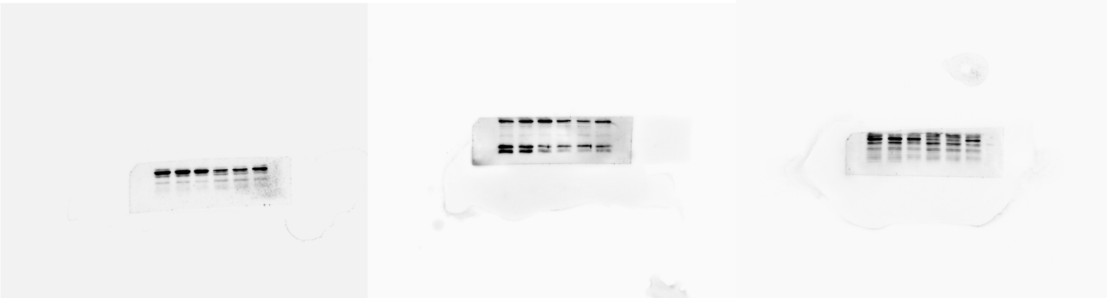

Caspase3:

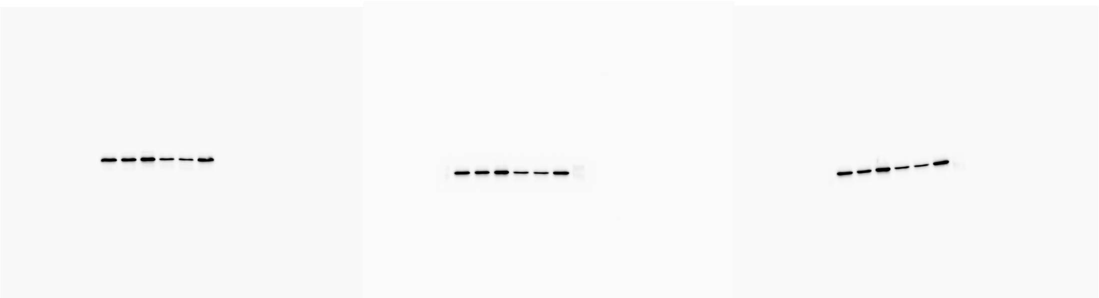

Caspase9:

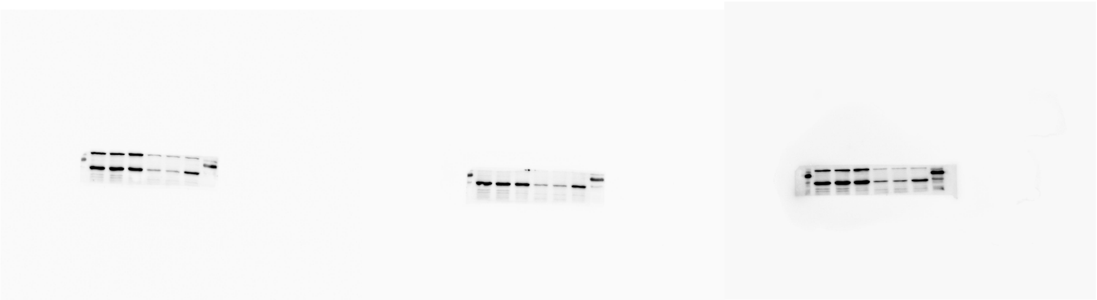

Cleaved-Caspase3:

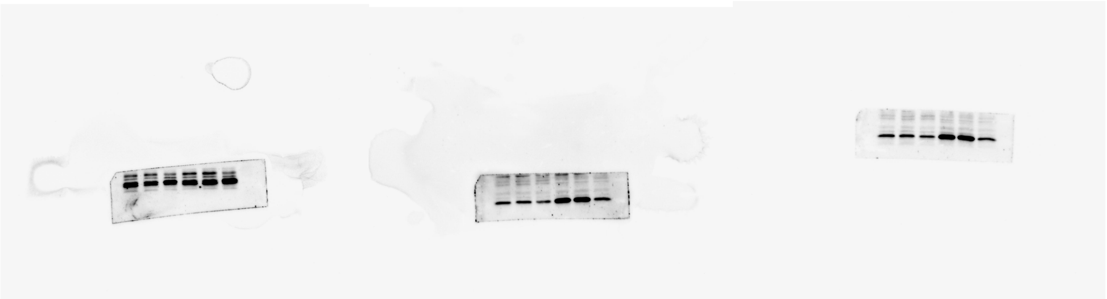

Cleaved-Caspase9:

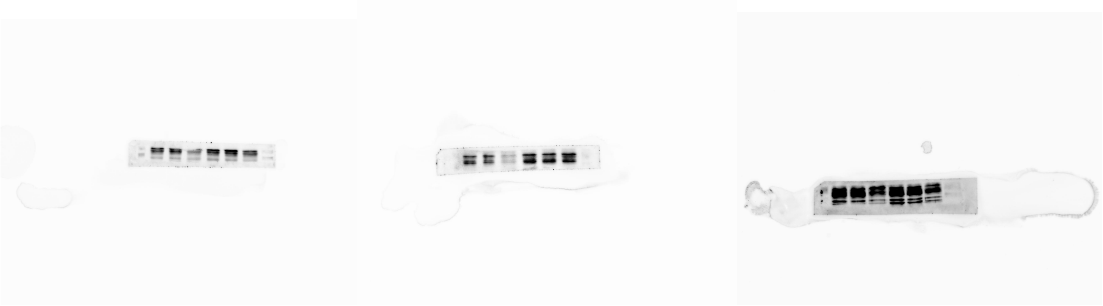

Figure 3D

FUNDC1:

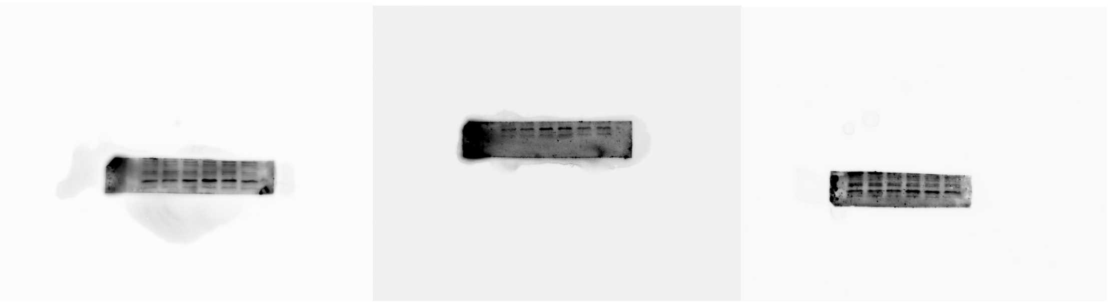

Figure 4F

Mito-Cytc:

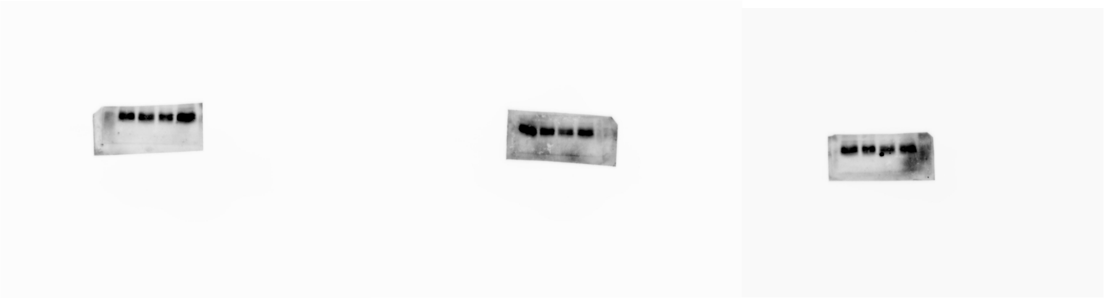

Cyto-Cytc:

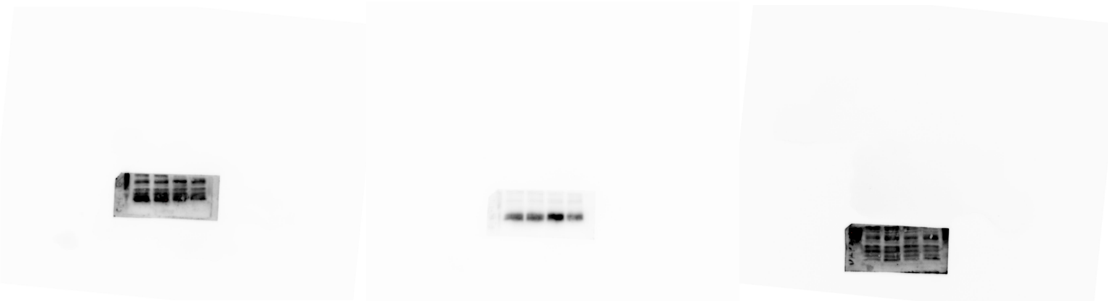

Figure 5D

LC3B:

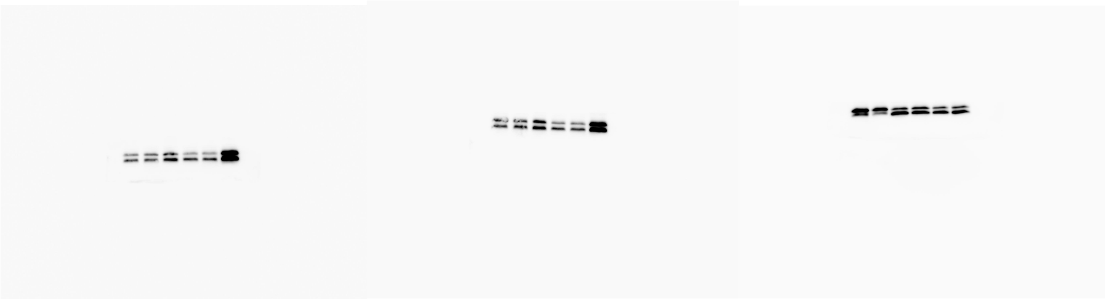

P62:

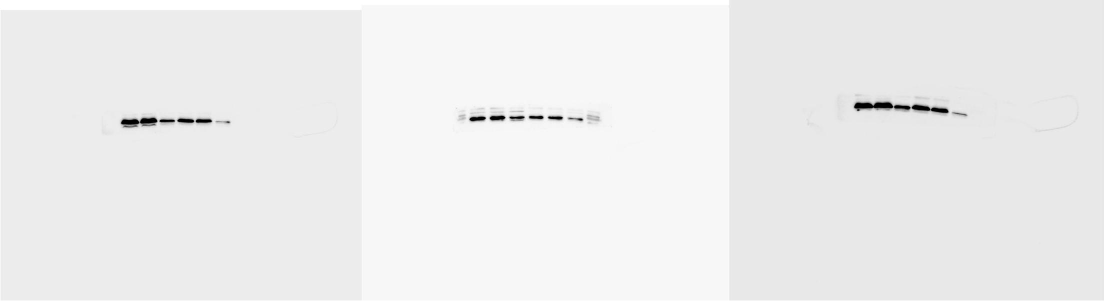

Figure 6A

Bax:

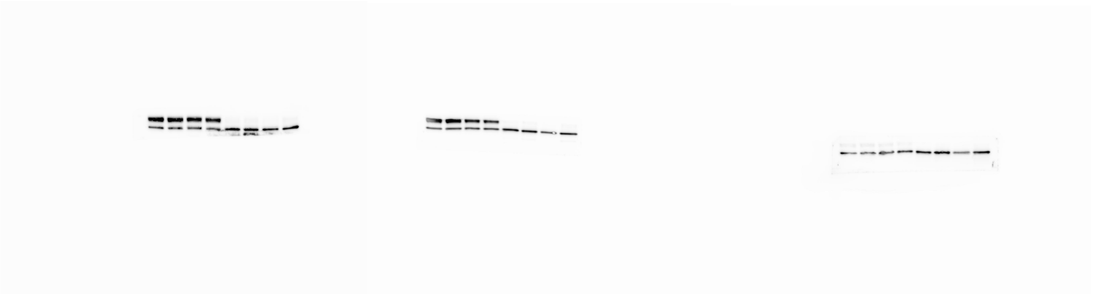

Bcl-2:

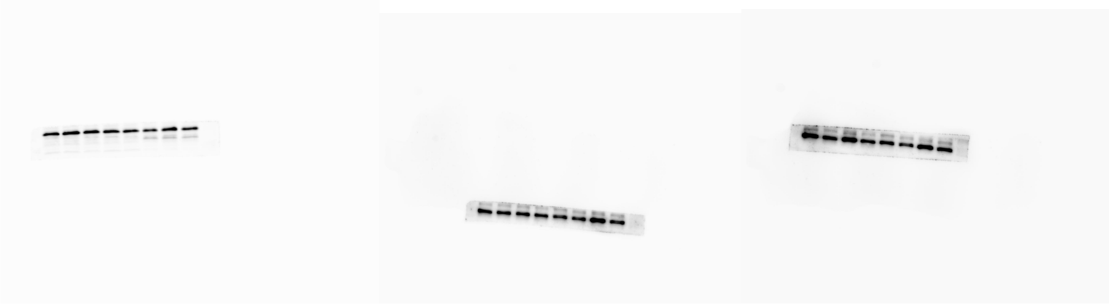

Cleaved-Caspase3:

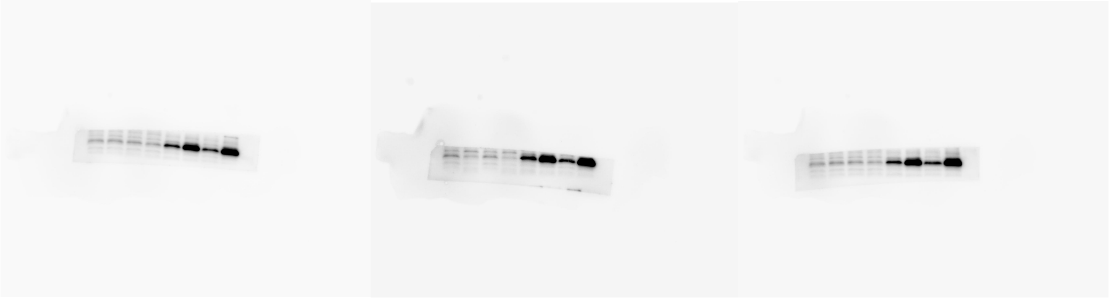

Cleaved-Caspase9:

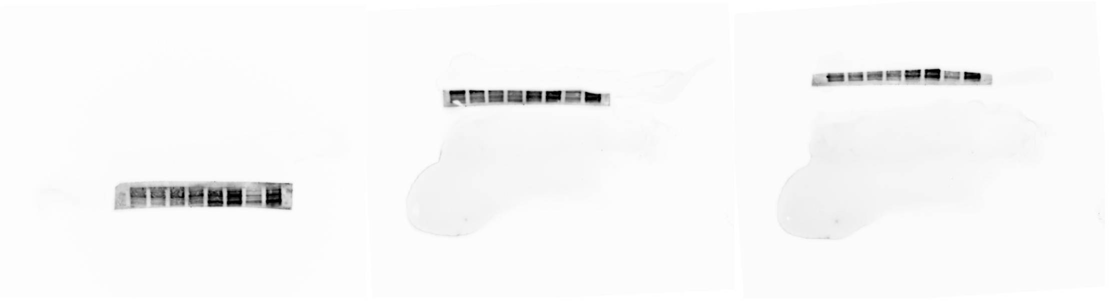

TIMM23:

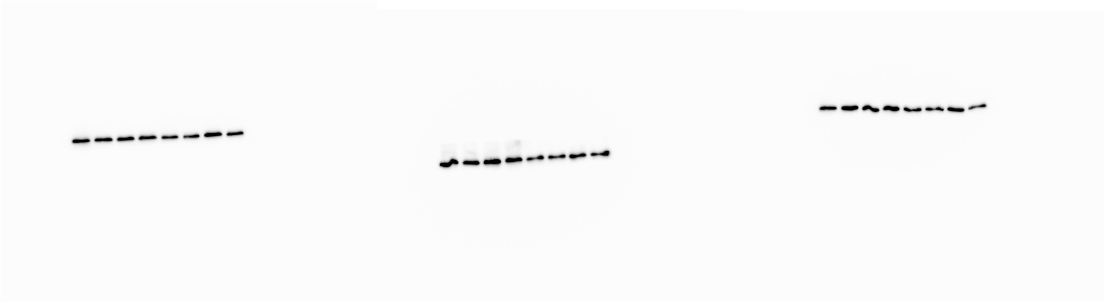

TOMM20:

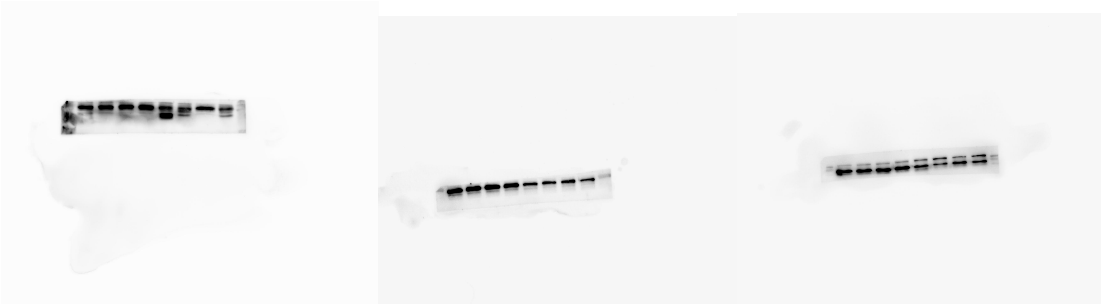

Figure S1

FUNDC1:

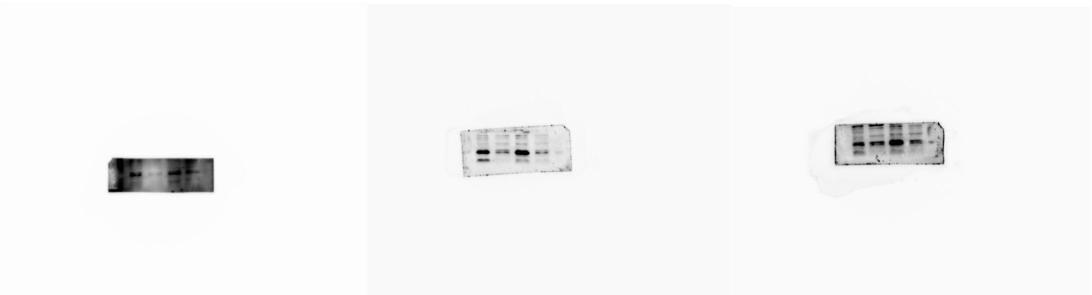

Bax:

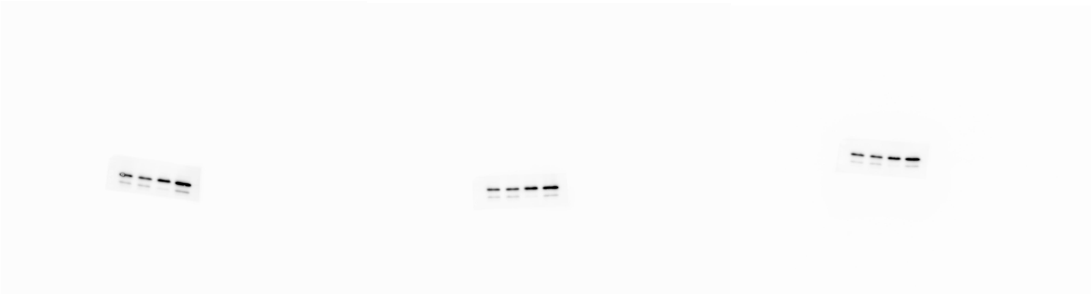

Bcl-2:

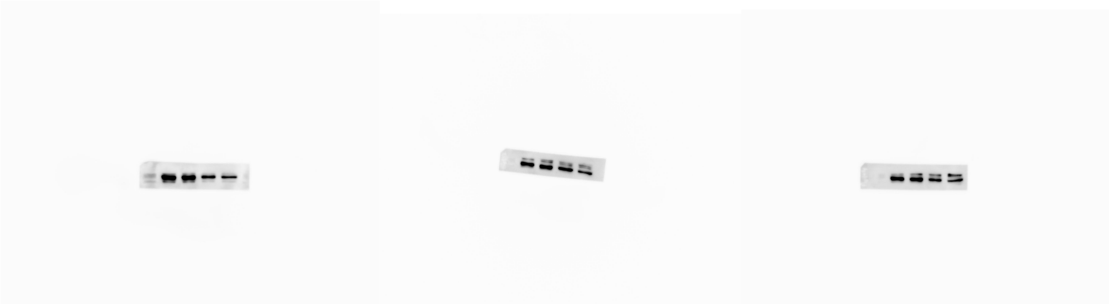

Cleaved-Caspase3:

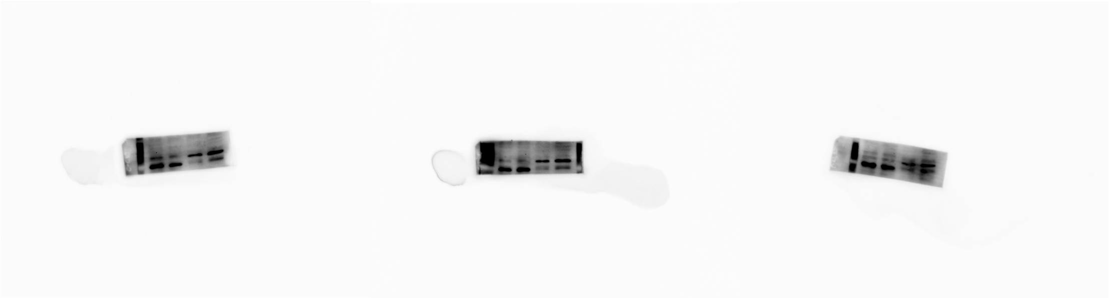

Cleaved-Caspase9:

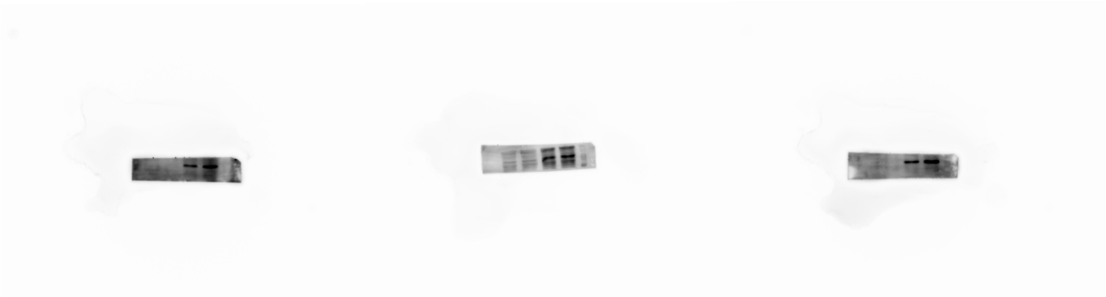

Supplement: Supplementary file 2 — Original Data File [file 41420_2023_1780_MOESM2_ESM.pdf]
